# Egocentric Temporal Order Bias Robust Across Manipulations of Cue Predictability and Sensory Modality

**DOI:** 10.1038/s41598-020-59912-5

**Published:** 2020-02-19

**Authors:** Ty Y. Tang, Michael K. McBeath

**Affiliations:** Department of Psychology, Arizona State University, Tempe, USA

**Keywords:** Cognitive neuroscience, Perception, Sensory processing, Human behaviour

## Abstract

The Egocentric Temporal Order (ETO) bias is the finding that self-initiated action-events are perceived as having occurred prior to simultaneous externally triggered events. Here, we test if the ETO bias is affected by predictability of the stimulus cue used to initiate a self-action or by the sensory modality of that cue. Without separating out the potential influence of the stimulus cue on the ETO bias, further investigations into the mechanisms underlying the bias are difficult to interpret. Our findings robustly confirm and replicate the ETO bias, providing evidence that the bias is not an artifact of the experimental design, but rather indicates a true temporal bias in the perception of self-initiated action-events.

## Introduction

One’s experience of reality requires constant temporal integration of sensory inputs. From lining up a touch and sound so that they feel simultaneous, to temporally binding an action and its delayed effect with causation, our brains are remarkably good at piecing together signals to construct a holistic perception of our world^1–6^. However, this temporal integration can oftentimes lead to illusions and misperceptions during narrow temporal intervals^7–15^.

In a recent study, we documented a bias for participants to perceive their self-generated events (in this case, touching a sensor) as occurring before simultaneous externally controlled events (such as being touched, or hearing an auditory beep)^15^. This bias, which we named the Egocentric Temporal Order (ETO) bias, provides insight into arguments about temporal order, especially in domains such as basketball and soccer, in which competing players often appear to code the perceived temporal order to their advantage. Our findings, in which observers systematically perceived their own self-initiated action events to occur earlier than external events, were consistent across stimulus modality comparisons and was independent of whether they were comparing their own touch against another person’s touch or a mechanical touch. However, in the previous study, all experimental manipulations used the same time-randomized cuing procedure to signal when to initiate the touch. Specifically, the stimulus cue for each experiment used an LED flash, randomly delayed between 200–1000 milliseconds. In the present study, we test if the ETO bias differs between conditions when the response cue is presented in a predictable manner that is likely to affect planning, or when the sensory modality of the cue is altered.

We originally examined the ETO bias in the context of making temporal order judgments in sports such as basketball and soccer, but in both sports, players are typically able to predict when they must initiate an action to contact the ball. That is to say, the stimulus cue to initiate an action is predictable, unlike in our initial study. This is important because when the cue is unpredictable, more attentional resources are required to perform the action and the temporal perception of the two events can be more susceptible to effects such as prior entry and inattentional blindness^16–23^. If increasing the cue predictability washes out the effect of the ETO bias, then the ETO bias may simply be an expression of attentional demand and prior entry.

In addition, in many cases, the stimulus cue to perform an action may be directed by another sense such as audition (for example, when a whistle is blown). The difference in cue modality may impact the ETO bias either due to differences in reaction times^24^ or differing perceptual durations^25^. This, in conjunction with perceptual illusions that may occur during integration and coupling of separate multisensory events, could lead one to perceive their partner’s touch to be temporally shifted^14,26–32^. If changing the stimulus cue from a visual flash to an auditory click significantly impacts the ETO bias, then the ETO bias may represent a perceptual illusion resulting from coupling or interference between the cue and external event.

In contrast, if the ETO bias is still present and unaffected by the temporal reliability or sensory modality of the cue, that supports the interpretation that the ETO bias is due to a generic temporal bias between the experience of self-initiated actions and unplanned external events.

## Methods

**Figure 1:**
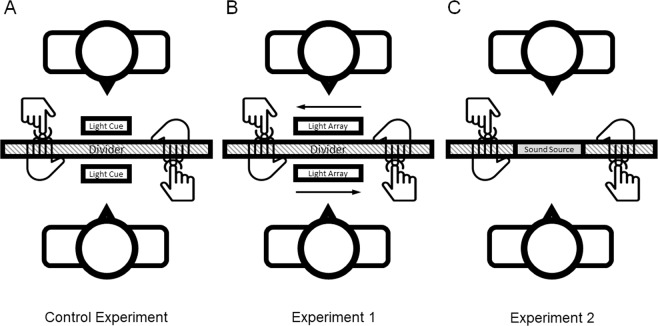
A cartoon illustration of the experimental setup. Participants sit across from one another with a visual divider between them. Both participants have capacitive sensors fixed to their left hands. When cued, participants use their right hand to touch the sensor on their partner’s left hand. In Experiment 1, participants are cued using an LED countdown moving from left to right relative to the participant, terminating in a flash. The LED array is 5 centimeters across and located approximately 60 centimeters away from the participant. In Experiment 2, participants are cued using an auditory click.

Experiments 1 and 2 both used modified versions of the experimental protocol developed for studying the ETO bias. This experimental method, which provides a control group for the present study, has a dyad of participants sit at a table across from one another with a visual divider in between them, and two holes to stick their hands through (Fig. 1A). Two blue LEDs are positioned on both sides of the divider and are electrically coupled. The LEDs serve as a synchronized stimulus cue for participants to touch a capacitive sensor on their partner’s hand. As they do so, a sensor on their own opposite hand is tapped by their partner. The LEDs are programmed to flash after a random interval between 200 and 1000 milliseconds after both participants indicate that they’re ready for the next trial. Trials where either participant touches before the cue is presented or when either touch occurs over 250 milliseconds after the cue are not recorded.

In Experiment 1, we increase the predictability of the LED cue to manipulate attentional demands of the task by changing the participant’s action from reactionary to predictable. Instead of the LED flashing at an unpredictable time, an additional array of 4 LEDs successively turns off, signaling a countdown to the stimulus cue flash (Fig. 1B). The LEDs begin counting from a random position, 1 to 4, terminating with a blue LED flash when the last LED of the array turns off. Each LED of the array stays on for 500 milliseconds, so the total countdown time ranges in discrete intervals from 500 milliseconds to a maximum of 2000 milliseconds. The LEDs are colored red, orange, yellow, green, and blue such that it always proceeds in that order from left to right, terminating with the blue LED flashing. This way, the blue LED flashing is always the cue for the participants to touch. Trials where participants touch either 250 milliseconds before the cue or 250 milliseconds after the cue are not recorded.

In Experiment 2, we manipulate the stimulus modality to test if sensory modality of the cue impacts the ETO bias. This experiment is identical to the control, except the light countdown is replaced by an auditory click (Fig. 1C). As in the control, the click occurs after a random interval of between 200 to 1000 milliseconds after both participants indicate they are ready for the next trial. Participants are instructed to touch the sensor on their partner’s hand when they hear the click. Trials where touches occur before the cue or 250 milliseconds after the cue are not recorded.

In all conditions, participants end each trial by making independent and anonymous temporal-order judgments on which haptic event (their touch or their partner’s touch) occurred first using a game controller. Synchronized timing data was collected through an Arduino Mega 2560 microprocessor board. Participants are not given feedback between trials and are instructed to not communicate with their partner. Each dyad performs 50 trials.

Participant data was excluded in cases where participants clearly did not follow directions, such as when a participant only used one response (always indicated one person touched earlier regardless of actual order), responded at random (no correlation between judgments and the actual touch times differences), or systematically responded incorrectly (their judgment was inversely correlated with actual temporal differences).

Data across all conditions were analyzed using a binary logistic regression model, comparing the temporal order judgment (“I touched first” vs “they touched first) to the actual time difference between the touches. Data were modeled per participant, and the ETO bias was compared between groups through an analysis of variance.

This study was approved by the Arizona State University Institutional Review Board Office. The study was performed in accordance with the ethical standards of the CITI Human Subjects Research guidelines. All participants provided an informed consent before participating in the study.

## Results

The data are analyzed per-participant using a binary logistic regression model. The data are represented with temporal difference as the independent measure and temporal order judgments as the dependent measure. In all cases, temporal differences were calculated by taking a participant’s touch timing and subtracting their partner’s touch timing. For example, if a participant’s touch occurs 550 milliseconds after the trial begins and their partner’s touch occurs 600 milliseconds after the trial begins, then temporal difference would be −50 milliseconds. In this way, negative temporal differences indicate that the participant was faster than their partner on that given trial, while positive temporal differences indicate that the participant was slower than their partner on that trial. The temporal order judgment represents a binary decision, where the manipulated stimulus is compared to the control stimulus. In this paradigm, a decision of “my touch occurred first” is coded as 0, whereas a decision of “their touch occurred first” is coded as 1. In this way, the *y* value of the regression model represents the probability of a participant judging their partner’s touch as having occurred first for a given time difference.

These data are then modeled per participant using a binary logistic regression model of the form:

$$y={(1+{e}^{-({\beta }_{0}+{\beta }_{1}x)})}^{-1}$$

where β_0_ is the y-intercept and β_1_ represents the shape of the function. In the context of our study, *x* represents the time difference between the two stimuli, *y* represents the probability of the temporal order judgment (TOJ), and β_0_, represents the probability in log space of a participant’s judgement with a 0 millisecond difference between stimuli (Bias). The model can therefore be rewritten as:

$$P(TOJ)={(1+{e}^{-(Bias+{{\rm{\beta }}}_{1}\ast \Delta Time)})}^{-1}$$

**Figure 2:**
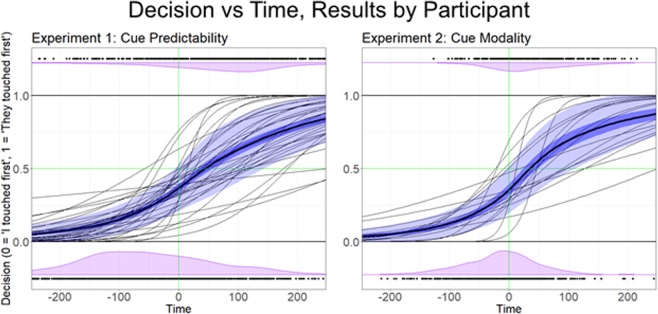
The binary logistic regression data from both experiments. The thin black lines represent individual binary logistic regression models for each participant. The bold black line is the regression across the entire group. The dark blue band represents one standard error, and the light blue band represents one standard deviation. Black dots on the top and bottom represent individual responses, with responses at the bottom representing trials where participants responded “I touched first”, and responses at the top representing trials where participants responded “they touched first”. The purple areas represent the distributions of those respective responses across time.

In each experimental condition, the ETO bias is captured by the statistical significance of the y-intercept of the model, indicating that the temporal-order judgment of two simultaneous events is not at chance level. Specifically, a positive value of the y-intercept means there is a bias toward perceiving their partner’s touch as having occurred first, and a negative value of the y-intercept means there is a bias toward perceiving their own touch as having occurred first. Binary logistic regressions for both experiments are shown in Fig. 2, along with distributions of individual participant responses.

In Experiment 1, using the model described above, we analyzed data from 35 undergraduate psychology students at Arizona State University. Across all 35 participants, we find a β_0_ coefficient of −0.630 (t(34) = −4.513, p < 0.001, d = −0.763). When converted from log space to probability space, this value corresponds to a 64.20% probability of participants believing their touch was first, even when both touches are simultaneous.

Additionally, we ran a separate model for Experiment 1 to see if the countdown time (500~2000 milliseconds) was a significant moderator of participant judgments, indicating that the ETO bias is affected by the degree of cue predictability:

$$P(TOJ)={(1+{e}^{-(Bias+{{\rm{\beta }}}_{1}\ast \Delta Time+{{\rm{\beta }}}_{2}\ast Countdown+{{\rm{\beta }}}_{3}\ast \Delta Time\ast Countdown)})}^{-1}$$

Using a Wald test, we found that neither the duration of the countdown (indicated by β_2_ in the above model) (z = 0.409, n.s.), nor the interaction between the countdown and time difference (β_3_ in the above model) (z = 1.041, n.s.), was a significant predictor of the temporal order judgment.

In Experiment 2, we analyzed 18 undergraduate psychology students at Arizona State University, and our binary logistic regression analysis revealed a β_0_ coefficient of −0.678, corresponding to a 66.42% probability of believing their touch happened first even when both touches were simultaneous (t(16) = −3.4428, p < 0.005, d = −0.835).

Finally, we wanted to know whether the results of Experiments 1 and 2 differed significantly from the unpredictable light cue of the original study. Using Experiment 1 from our previous study as a control condition, we conducted an analysis of variance (ANOVA) between the ETO bias of each condition and found no significant differences between the experiments (F(2, 66) = 0.2714, n.s.). However, due to the nature of null hypothesis testing, a Bayesian factor analysis was conducted to validate the result^33–36^. By comparing the ratio of the probability of the null model (temporal order judgments are the same between conditions) against the probability of the alternative model (temporal order judgments are different between conditions), we attain Scaled JZS Bayes Factors^34,37^ of 4.415 and 3.945 for Experiments 1 and 2, respectively. These results further support the null finding from ANOVA, confirming no statistically significant differences between conditions^33,35,36^ (R package ‘BayesFactor’ available from pcl.missorui.edu/bayesfactor).

**Figure 3:**
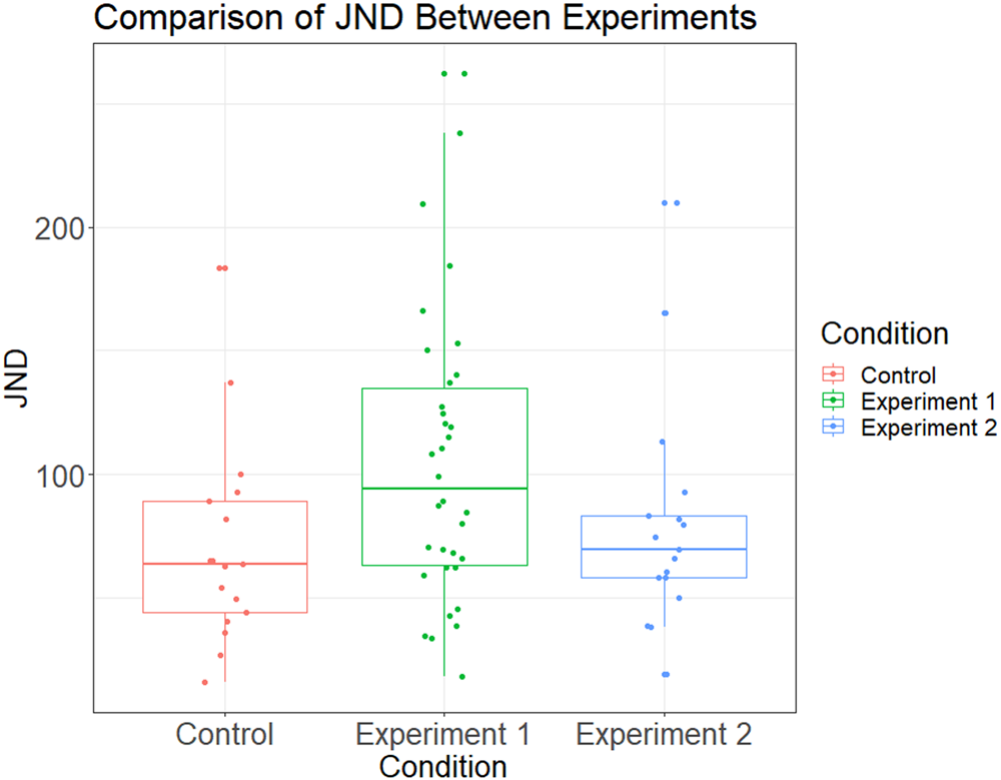
A box-plot comparing JNDs across conditions. Individual dots represent the JND for a particular participant in that condition. An analysis of variance reveals no significant differences between JNDs across experiments (F(2, 48) = 2.102, p = n.s.). However, a Bayesian factor analysis for Experiment 1 (shown in green) shows a significant difference in participant JND as compared to the control (t(49) = 2.146, p < 0.05). This corresponds to a higher sensitivity in judgment with respect to timing differences between the two stimuli.

**Figure 4:**
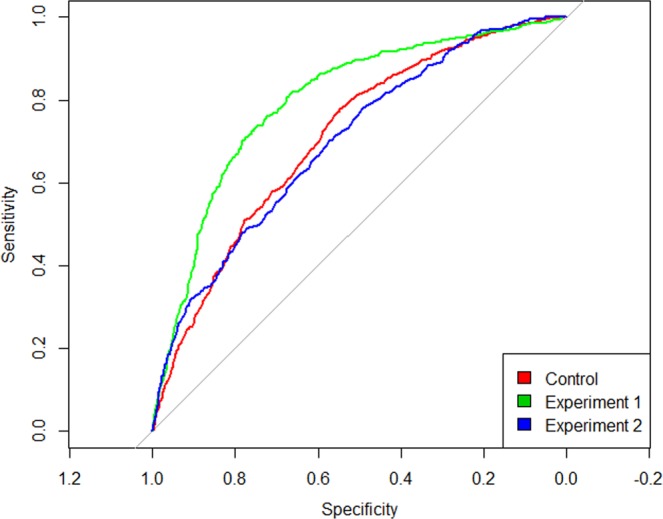
A Receiver Operating Characteristic curve of sensitivity vs specificity across all 3 conditions. Specificity is calculated as the true positive rate divided by the sum of true positives and false negatives (TP/TP + FN)), and sensitivity is calculated as the false positive rate divided by the sum of false positives and true negatives (FP/(FP + TN)). In the case of this data, true negatives are trials where participants judge their touch to happen first when it actually happened first, and true positives are trials where participants judge their partner’s touch to happen first when it actually happened first.

In a post-hoc test, we examined whether sensitivity was significantly different across experiments by looking at the just noticeable difference (JND) between experiments. We used the individual participant regression models to calculate the JND using the method of constant stimuli^38,39^ (Fig. 3). When comparing participant JNDs across experiments through ANOVA, we found no significant differences (F(2, 48) = 2.102, p = n.s.). However, a Bayesian Factor Analysis does reveal evidence for a difference in JND between Experiment 1 and the control (t = −2.146, Scaled JZS Bayes Factor = 1.82), but not for Experiment 2 (t = −0.585, Scaled JZS Bayes Factor = 2.663), indicating that participant sensitivity is higher in Experiment 1, compared to the control. This difference is reflected visibly in a Receiver Operating Characteristic curve (Fig. 4). Data are provided as supplementary information and code used for analysis may be provided upon request (Supplementary Dataset 1).

## Discussion

**Figure 5:**
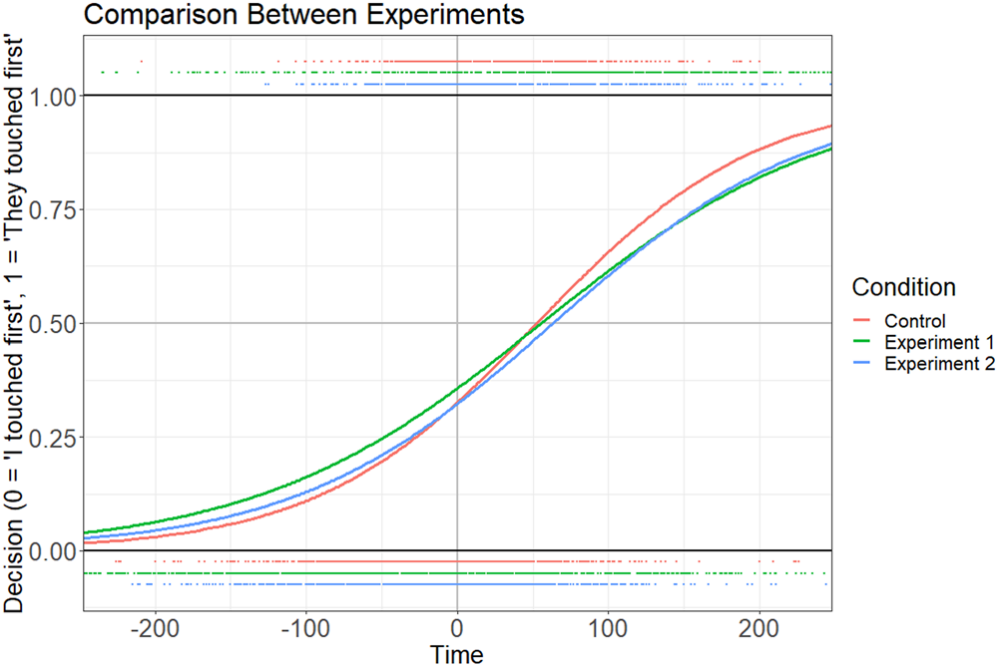
A comparison of the binary logistic regression models between the two experimental groups and control group. Colored dots on the top and bottom of the graph indicate individual responses for that group, corresponding to responses “they touched first” and “I touched first”, respectively. Individual responses have been vertically offset from 0 and 1 for visual clarity. The regression curve represents the probability of a participant responding “their touch happened first” at any given time difference. Negative times indicate trials where the participant touched earlier than their partner and positive times indicate trials where the participant touched after their partner.

**Figure 6:**
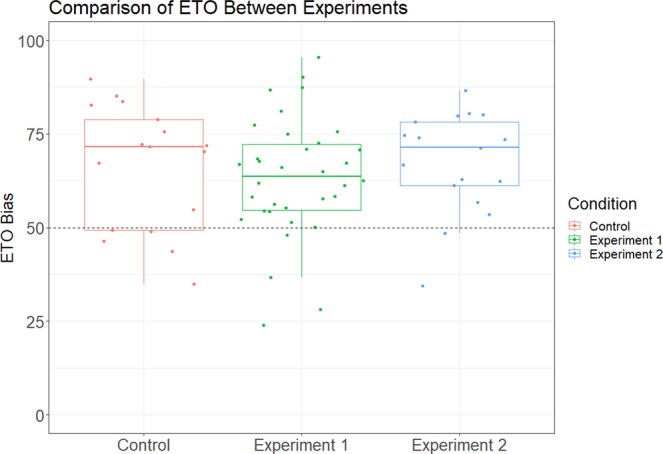
A box-plot showing the ETO biases for participants across the two experimental groups and control group. The 50% threshold is highlighted to indicate equal chance of responding “I touched before” or “I touched after” when both touches are simultaneous. Values have been converted from log space to probability space for ease of interpretation.

Across both cue manipulations, we replicate the ETO bias: participants’ temporal order judgments are significantly biased to judge their own touch as happening first (Figs. 5 and 6). When increasing cue predictability with a count down, participants are biased to perceive their touch as happening before another simultaneous touch 64.20% of the time. Additionally, we find that the length of the countdown is not a significant predictor of temporal order judgments. When the cue stimulus is changed to an unpredictable auditory click, participants perceived their touch as happening first 66.42% of the time. An analysis of variance and Bayesian factor analysis reveal no significant differences between the two experimental manipulations and the control condition^34^.

Our main finding, that the ETO bias is present across manipulations of both the cue’s temporal predictability and stimulus modality, provides evidence that the ETO bias is not simply an effect of experimental design. The Bayesian analysis supports that the cue manipulations do not significantly influence the presence or magnitude of the ETO bias, further supporting that the ETO bias represents a perceptual difference in the two sensory events. This analysis is particularly important in providing evidence that the significant biases seen in the cue manipulations are indeed manifestations of the ETO bias, rather than unrelated significant effects from differences in cue manipulation^33^. It is worth noting that while manipulating the predictability of the cue does inherently increase predictability of the sensory event, it does not rule out attention or predictability of the sensory event itself as an underlying cause of the ETO bias. Indeed, further research is required to elucidate whether the ETO bias could be driven by mechanisms such as intentional binding^2,3^ or prior entry^17,23^. However, the present study’s findings, that the ETO bias is not driven by the predictability or sensory modality of the cue, represents an integral prerequisite step to interpreting evidence regarding the ETO bias.

Finally, the present study contributes to a larger understanding of how vantage can systematically bias perceptual experience^7,13,15,40–42^. More broadly, we hope that in time, such findings will cue individuals to consider how differences in judgment may arise from two differing vantages of the same situation, as well as how their own beliefs and experiences have been shaped by their unique perspective.

### IRB protocol

This study, STUDY00006929, was approved by IRB coordinator E. Williams of the Arizona State University Institutional Review Board Office. Both authors have completed Human Research: IRB–Social & Behavioral Research (Group 2) CITI training and hold active certificates.

## Supplementary information

Supplementary Dataset 1.
